# Fabrication and Characterization of a Novel Smart-Polymer Actuator with Nanodispersed CNT/Pd Composite Interfacial Electrodes

**DOI:** 10.3390/polym14173494

**Published:** 2022-08-26

**Authors:** Jie Ru, Dongxu Zhao, Zicai Zhu, Yanjie Wang

**Affiliations:** 1 Key Laboratory of Green and Precise Synthetic Chemistry and Applications, Ministry of Education, School of Chemistry and Materials Science, Huaibei Normal University, Huaibei 235000, China; 2 Jiangsu Key Laboratory of Special Robot Technology, Hohai University-Changzhou, Changzhou 213022, China; 3 College of Mechanical and Electrical Engineering, Inner Mongolia Agricultural University, Hohhot 010018, China; 4 School of Mechanical Engineering, Xi’an Jiaotong University, Xi’an 710049, China

**Keywords:** smart polymer, IPMC, composite electrode, electrochemical characteristics, actuation behavior

## Abstract

As emerging smart polymers, ionic polymer-metal composites (IPMCs) are playing more and more important roles as promising candidates for next-generation actuators in terms of academic interest and industrial applications. It is reported that the actuation behaviors of IPMCs are dependent on the electrochemical kinetic process between metal/polymer interfaces to a great extent. Thus, the fabrication of tailored metal/polymer interface electrodes with large surface areas and superior interface characteristics is highly desirable in improving the actuation performance of IPMCs, which is still technologically critical for IPMCs. In this contribution, we developed a novel fabrication technology for carbon/metal composite electrodes with a superior interface structure and characteristics to optimize the actuation behaviors of IPMCs by exploiting the synergistic effect of combining a sulfonated multi-walled carbon nanotube (SCNT)/Nafion hybrid layer with nanodispersed Pd particles. The improved IPMCs showed significantly enhanced capacitance characteristics and highly facilitated charge–discharge processes. Moreover, their actuation behaviors were greatly improved as expected, including approximately 2.5 times larger displacement, 3 times faster deformation speed, 4 times greater output force, and 10 times higher volume work density compared to those of the IPMCs with traditional electrode structures. The advantages of the developed SCNT/Pd-IPMCs will greatly facilitate their applicability for artificial muscles.

## 1. Introduction

As typical smart polymers, ionic polymer-metal composites (IPMCs) have attracted great attention as promising candidates for next-generation actuators in bionic actuation fields [1,2]. Usually, an IPMC has a metal electrode-polymer electrolyte-metal electrode configuration, consisting of an ionomer membrane (such as Nafion, Flemion, etc.) and mobile hydrated cations sandwiched between two metal electrodes (such as gold and palladium, etc.). When submitting to an electric field, the hydrated cations in the polymer electrolyte migrate to the cathode side by means of electrostatic forces [3,4]. As a result, the IPMC shows a bending deformation toward the anode side, resulting in actuation capabilities [5,6,7] (as shown in Figure S1 in the Supplementary Materials). The actuation capabilities of IPMCs facilitate their promising applications in artificial muscles [8,9,10]. However, some of the major drawbacks of IPMCs (such as relatively low carrying capacity, slow deformation speed, and relaxation deformation) seriously restrict the engineering applications of conventional IPMCs.

In order to meet the requirements of engineering applications, more and more efforts have been devoted to improving the actuation capabilities of IPMCs. Some researchers have improved the chemical and mechanical characteristics of IPMCs by synthesizing novel ionomer membranes [11,12,13] and doping membranes with nanoparticles [14,15]. Meanwhile, some researchers have optimized the electrochemical and electromechanical properties of IPMCs by thickening the electrodes via multiple plating or additional deposition [16,17,18,19,20,21], and employing novel materials (such as nanocarbon materials and conducting polymers) [22,23,24]. Despite the achieved progress, most of the previous demonstrations have not been able to achieve large deformation and high output force simultaneously.

In fact, the electrode of an IPMC has two distinct layers, including a surface electrode and a metal/polymer interface electrode. It is reported that the actuation capabilities of IPMCs are dependent on the electrochemical kinetic process between the metal/polymer interface for charge accumulation to a great extent, as the actuation performances are directly related to the capacitance properties [25,26,27,28]. A large specific surface area of the electrode can give rise to a double-layer capacitance of IPMCs, and then result in superior actuation capabilities [29,30]. Thus, it would be a reliable and simple approach to simultaneously improve the actuation capabilities of IPMCs via manipulating and optimizing the interface structure and characteristics. Accordingly, many researchers have committed to adjusting the characteristics of specific nanostructures to create metal/polymer interfaces with large surface areas, in order to realize a large contact area and superior electrochemical and electromechanical performances for IPMCs [31,32,33,34]. Previous approaches have attempted to increase the surface area by simply thickening the electrode via repeated plating or additional deposition [17,19,29,35], and developing metal/non-metal composite electrodes by physical methods [36,37]. However, such approaches usually have little effect on increasing the penetration depth of metal electrodes. Thereby, they presumably could not obtain optimum interface electrodes. Thus, such demonstrations may not assist in enhancing the actuation capabilities of IPMCs effectively. Hence, the fabrication of a tailored interface electrode microstructure with a large surface area and superior characteristics is still technologically critical and highly desirable in updating the actuation capabilities of IPMCs.

**Figure 1:**
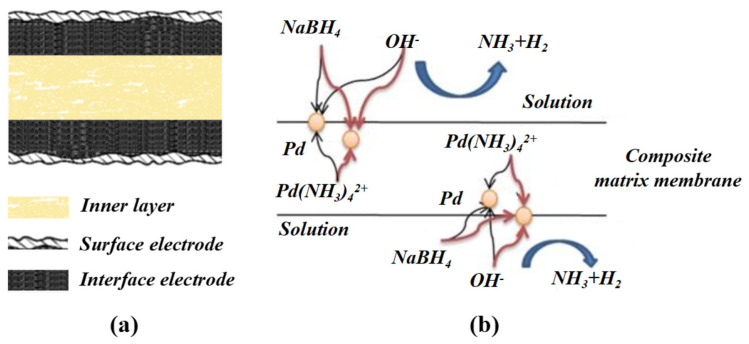
(**a**) Schematic illustration of SCNT/Pd−electrode IPMC; (**b**) formation mechanism of SCNT/Pd composite interface electrode.

To our knowledge, the actuation capabilities of IPMCs can be enhanced to a certain degree by incorporating nanocarbon materials into the electrolyte membrane [1,14,38,39]. In our previous research, it was found that the ionic exchange capacity (IEC) and mechanical properties of a Nafion membrane can be greatly increased by doping sulfonated multi-walled carbon nanotubes (SCNTs) [38]. In addition, the SCNTs would serve as a large number of adsorption sites for metal complex cations, since the SCNTs have quite large specific surface areas [40,41]. Scanning electron microscopy (SEM) images have revealed that Nafion membranes doped with high contents of CNTs are filled with randomly oriented three-dimensional micropores [39]. In the impregnation-reduction plating (IRP) process, the large number of adsorption sites, high IEC, and porosity of the SCNT/Nafion hybrid layer can greatly facilitate the ion exchange of metal complex cations (like [Pt(NH_3_)_4_]^2+^ and [Pd(NH_3_)_4_]^2+^) and penetration of the reducing agent (like BH_4_^−^) into the matrix membrane, allowing the formation of nanodispersed metal particles inside the IPMC. Based on the above findings, we propose to introduce a high content of SCNTs into the Nafion layer, combined with the IRP process, to form SCNT/Metal (SCNT/M) composite interface electrodes in IPMCs. This would create an SCNT/M composite interface electrode with a strong synergistic effect by exploiting the SCNT/Nafion hybrid layer and nanodispersed metal particles together if they are compatible with each other. Figure 1 shows the schematic illustration of SCNT/Pd−electrode IPMC and formation mechanism of SCNT/Pd composite interface electrode. The large surface area and superior capacitance of the composite interface electrodes are favorable to accelerate ion transport and accumulate hydrated cations under an electric field [42,43], especially in the SCNT/Nafion layer containing a large number of hydrated cations. Moreover, the increase in the stiffness of the IPMC by doping with a high content of SCNTs in the composite interface electrodes would also enlarge the output force of the actuator. As a result, the formation of an SCNT/M composite interface electrode is expected to give rise to sufficiently large deformation and high output force simultaneously.

In our previous research, we employed relatively inexpensive [Pd(NH_3_)_4_]Cl_2_ instead of Au and Pt compounds to develop a new kind of IPMC with Pd electrodes [4,11,22,32,38,44]. The performances of the Pd-electrode IPMCs are comparable to those of the Pt-electrode IPMCs [6,9,13,17,19,30]. Thus, in this research, we chose Pd as the metallic electrode. Actually, we took the preparation of an SCNT/Pd composite electrode as an example to further study the technological method of developing a series of CNT/Metal composite electrodes, such as CNT/Au, CNT/Pt, and CNT/Ag composite electrodes, in the forthcoming works.

Herein, we exploit a novel and effective route to develop thickness-controlled nanodispersed SCNT/Pd interface electrodes to achieve significant enhancements in both the deformation and output force of IPMCs. Firstly, a Nafion layer sandwiched between two SCNT/Nafion hybrid layers was hot-pressed to prepare the composite matrix membrane. Secondly, nanodispersed Pd particles were plated on both sides of the composite matrix membrane by IRP to form SCNT/Pd composite interface electrodes. Thirdly, the Pd surface electrode was deposited by autocatalytic plating to decrease the surface resistance of the resulting IPMCs. Finally, the electrochemical and electromechanical properties were measured to evaluate the reinforcing effects of the composite interface electrodes in the actuation capabilities of the IPMCs systematically.

## 2. Materials and Methods

### 2.1. Experimental Materials

The perfluorosulfonic acid (PFSA) resin Nafion dispersion (DE-520, 5.0–5.4 wt% in a mixture of propanol and water) was purchased from DuPont Company (Washington, WA, USA). Pd(NH_3_)_4_Cl_2_, NaBH_4_, N_2_H_4_·H_2_O, and ethylene glycol (EG) were purchased from Aldrich (Shanghai, China). The SCNTs used in this work were the same as those synthesized and used in our previous research [38]. Characterization analyses of the SCNTs are shown in the Supporting Information.

### 2.2. Preparation of SCNT/Nafion Composite Matrix Membrane

An SCNT/Nafion hybrid layer consisting of 10 wt% SCNT and 90 wt% Nafion was prepared as follows. A gelatinous mixture composed of 40 mg of SCNTs, 7.2 g of Nafion dispersion, and 4 g of EG was prepared via sonication treatment for 30 min and stirring for 4 h, successively. The above mixture was cast in a PDMS mold (40 mm × 60 mm × 40 mm) by evaporating the solvent at 90 °C for 24 h, and annealing at 120 °C for 1 h, successively. The thickness of the obtained SCNT/Nafion hybrid layer was 80–90 μm. A Nafion inner layer with a thickness of 175–185 μm was prepared by casting a mixed solution composed of 18 g of Nafion and 4 g of EG in a PDMS mold (40 mm × 60 mm × 40 mm) using the same solvent evaporating process. The composite matrix membrane was fabricated by hot-pressing the Nafion inner layer sandwiched between two SCNT/Nafion hybrid layers with a pressure of 4 kgf/cm^2^ under 180 °C for 180 s. For comparison, a pure Nafion layer and a pure Nafion matrix membrane without SCNTs were also prepared using the same method.

### 2.3. Fabrication of IPMCs

**Figure 2:**
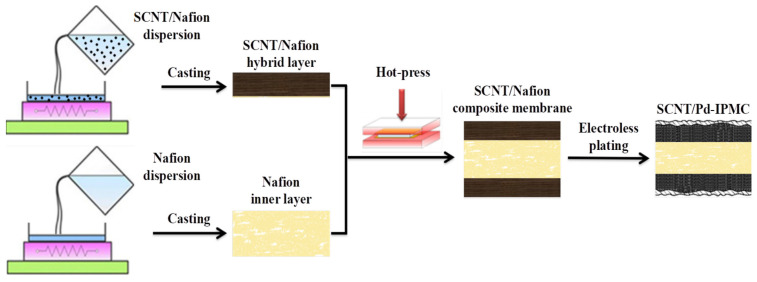
Schematic illustration of fabrication process of SCNT/Pd-IPMC.

The IPMCs were fabricated by assembling the matrix membranes and Pd electrodes via four successive steps, comprising surface roughening treatment, impregnation-reduction plating, autocatalytic plating, and actuating ion exchange. (1) In the surface roughening treatment, the matrix membranes were roughened with a sandblasting machine to increase the adhesion of the electrodes, then washed with ethanol solution and boiled in HCl solution and DI, successively. (2) In the IRP process, the pre-treated membranes were soaked in Pd(NH_3_)_4_Cl_2_ solution to exchange [Pd(NH_3_)_4_]^2+^ firstly, and were subsequently immersed in NaBH_4_ solution to plate nanodispersed Pd particles. It should be noted that the Pd atoms produce granular growth and agglomeration along the thickness direction, and further form SCNT/Pd composite interface electrodes by combining with SCNTs in the SCNT/Nafion hybrid layers. (3) In the autocatalytic plating (AP) process, Pd surface electrodes were deposited on both sides of the IPMCs, in order to decrease the surface resistance. (4) In the actuating ion-exchange process, Li^+^ was exchanged by soaking the IPMCs in LiOH/LiCl solution. Figure 2 represents schematic illustration of fabrication process of SCNT/Pd-IPMC. The size of the IPMC strips for characterization was 35 mm × 5 mm × (360 ± 10) μm under hydrated conditions. The detailed manufacturing process refers to our previous report [44].

### 2.4. Characterizations

The membranes were characterized by measuring the water uptake ratio (WUR) using the difference method, and ionic exchange capacity (IEC) using the acid-base titration method, with KOH (aq) and HCl (aq) [38], respectively. WUR was calculated according to Equation (1), while IEC was calculated according to Equation (2) [45].

(1) $$WUR=\frac{M-m}{m}\times 100\;(\%)\;$$

(2) $$IEC=\frac{V\times N}{m}\times 1000\;$$

where *M* is the mass (g) of the membrane in its hydrated state, *m* is the mass (g) of the membrane in its dry state, *V* is the amount (mL) of KOH (aq), and *N* is the normality (mol) of KOH (aq).

The cross-section morphology of the membranes and IPMCs was observed using field emission FE-scanning electron micrography (Zeiss Genimi SEM 500, Jena, Germany).

The electrochemical and capacitance characteristics of the IPMCs were analyzed using cyclic voltammetry (CV) and electrochemical impedance spectroscopy (EIS) techniques using a potentiostat VersaStudio (VersaSTAT 3, Princeton, NJ, USA) [38]. The details are described in the Supplementary Materials.

The bending stiffness (E) of the IPMCs was measured using the free oscillation attenuation method [32], and calculated according to Equation (3):

(3) $$E={\left(\frac{2\pi }{3.52}\right)}^{2}\frac{mf^{2}l^{3}}{I}=3.87\frac{\pi^{2}mf^{2}l^{3}}{ht^{3}}$$

where *E* is the equivalent elastic modulus (MPa), *I* is the moment of inertia (which is equivalent to *ht*^3^), *m* is the mass (kg) of the free end in the configuration, *f* is the natural frequency (Hz), *l* is the free length (m), *h* is the width (m), and *t* is the thickness (m).

The actuation capacities of the IPMCs were characterized by testing the displacement with a laser displacement sensor (Keyence, LK-G80, Osaka, Japan) and the blocking force with a microforce transducer (Transducer Techniques, GSO-10, Temecula, CA, USA) at a measuring distance of 20 mm with a free length of 30 mm [46]. In order to eliminate the effect of relaxation deformation under hydrated conditions on the test results of electrical and mechanical performances, the IPMCs were placed in the air for 100 s before testing. Three specimens were parallelly tested in each characterization.

## 3. Results and Discussion

### 3.1. WUR and IEC of the Membranes

**Figure 3:**
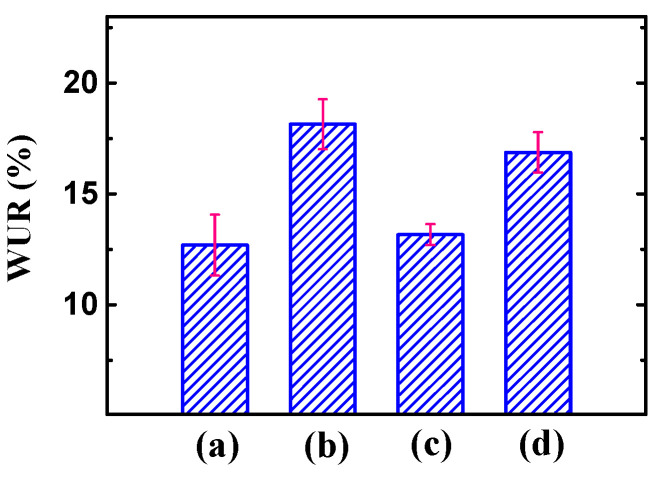
WUR of the prepared membranes. (**a**) Pure Nafion layer; (**b**) SCNT/Nafion hybrid layer; (**c**) pure Nafion matrix membrane; (**d**) SCNT/Nafion composite matrix membrane.

According to vehicle mechanisms and the Grotthus ‘hopping’ theory, WUR and IEC of the electrolyte layers are crucial factors that strongly affect the hydrated cation migration in terms of the mechanical strength and actuation performances of IPMCs [25,47]. As shown in Figure 3, the WUR values of the SCNT/Nafion hybrid layer and composite matrix membrane were 18.14% and 16.87%, while the WUR values of the pure Nafion layer and matrix membrane were 12.69% and 13.16%, respectively. In general, the WUR values of the SCNT/Nafion hybrid layer and composite matrix membrane were significantly improved by incorporating SCNTs, which is in contrast to the decrease in WUR of other Nafion-nano-filler systems such as MWNT-Nafion [38,48] and graphene-Nafion [49]. It is well known that MWCNT and graphene are hydrophobic particles. Substitution of Nafion with MWCNT or graphene would lead to an apparent decrease in the water absorption ability of the resulting membrane. In this research, hydrophilic -SO_3_H groups were grafted on the surface of SCNTs in the sulfonation process. The -SO_3_H groups provide sites of hydrogen bonding between SCNTs and water, giving excellent hydrophilicity and dispersibility to the SCNTs, which can be identified by the dispersity analysis of the SCNTs in Figure S3 in the Supporting Information. What is more, the superior hydrophilicity of SCNTs can greatly enhance the water absorption capacity of the Nafion membrane in the doping process. As a result, there was a significant increase in the WUR of the SCNT/Nafion hybrid layer and the composite matrix membrane.

In IEC testing, the SCNT/Nafion hybrid layer showed a dramatically improved IEC (2.48 mmol/g), which approximately increased by 2.1 times by comparison to that of the pure Nafion layer (0.79 mmol/g). The effective enhancement in IEC is mainly attributed to the great proton exchange ability of -SO_3_H groups grafted on the surface of SCNTs. In addition, the enhancement in WUR and IEC of the hybrid membranes may be due to increasing the size of ion clusters and the number of ion exchange sites for each cluster by incorporating SCNTs into the Nafion matrix [50]. The superior IEC and the large number of adsorption sites of the SCNT/Nafion hybrid layer would greatly facilitate the ion exchange of [Pd(NH_3_)_4_]^2+^ into the matrix membrane to form nanodispersed Pd particles for the SCNT/Pd-IPMC in the IRP process. What is more, the improvement of WUR and IEC of the hybrid membranes will result in superior actuation performances (including large bending deformation and high blocking force) of the corresponding actuators because of the movement of hydrated cations and water molecules under an electric field inside the actuators.

### 3.2. Cross-Section SEM Images of the Membranes and IPMCs

**Figure 4:**
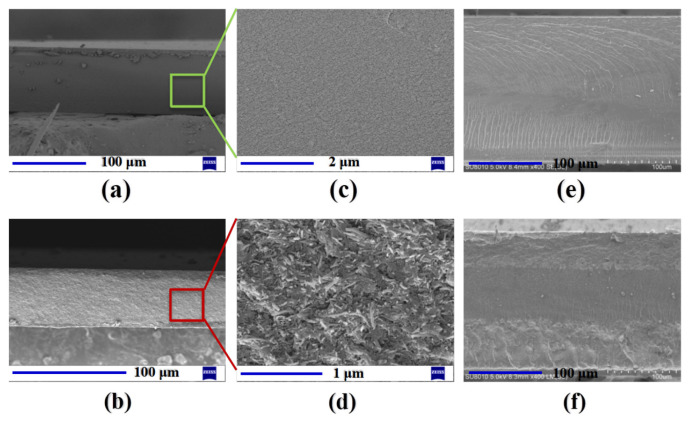
Cross-section SEM images of the membranes. (**a**) Pure Nafion layer; (**b**) SCNT/Nafion hybrid layer; (**c**) magnification level of pure Nafion layer; (**d**) magnification level of SCNT/Nafion hybrid layer; (**e**) pure Nafion matrix membrane after hot-process; (**f**) SCNT/Nafion composite matrix membrane after hot-process.

Cross-section SEM images of the pure Nafion layer and SCNT/Nafion hybrid layer before hot-pressing are represented in Figure 4a–d. It can be clearly observed that the pure Nafion layer possessed a typically smooth polymer surface with several wrinkles. Compared with the pure Nafion layer, the SCNT/Nafion hybrid layer showed a much rougher surface with a large amount of microchannels at the micron level, while the SCNTs were well enveloped in the Nafion matrix without any obvious agglomeration or entangled structure. The uniform dispersion of SCNTs contributed to their hydrophilic nature, which greatly facilitated the dispersion in the Nafion matrix through hydrophilic interaction between -SO_3_H groups of SCNTs and Nafion [38]. The large amount of microchannels of SCNT/Nafion hybrid layers, combined with their high IEC, will greatly facilitate the ion exchange of [Pd(NH_3_)_4_]^2+^ and penetration of BH_4_^−^ into the matrix membrane in the IRP process, which will allow the formation of nanodispersed metal particles inside the IPMCs.

The cross-section micrographs of the fabricated matrix membranes in Figure 4e,f clearly show that all three components were compactly adherent to each other without any wrinkles or gaps after hot-pressing. The pure Nafion matrix membrane has no distinguishable boundary between the pure Nafion layers and inner Nafion layer, which looks the same as those of traditional Nafion-117 membranes [1,14]. Conversely, there are distinct interleavers between the SCNT/Nafion hybrid layers and inner Nafion layer in the composite matrix membrane, which is due to the high content of SCNTs in the hybrid layer. The typical thickness of the obtained matrix membranes was around (330 ± 10) μm, which is smaller than the summation of three components. This is mainly because of the reduction in the thickness of each layer and interpenetration of layers caused by hot-pressing.

**Figure 5:**
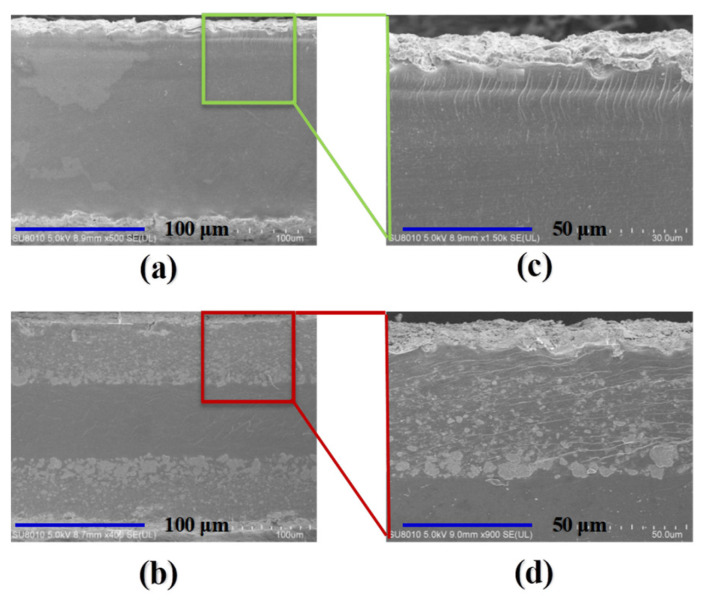
Cross-section SEM images of the IPMCs. (**a**) Pd-IPMC based on pure Nafion matrix membrane; (**b**) Pd-IPMC based on SCNT/Nafion composite matrix membrane, (**c**,**d**) magnification level of electrode and interface layer.

Figure 5a–d represent the architecture of Pd-IPMC and SCNT/Pd-IPMC based on the pure Nafion matrix membrane and SCNT/Nafion composite matrix membrane. For the Pd-IPMC, Pd particles were mostly located at the surfaces and the intermediate layer containing isolated nanoparticles was relatively thin (ca. 10–20 μm thick), which was the same as those of traditional Pd-IPMCs based on a Nafion-117 membrane [25,32,38]. In contrast, the electrode morphology of the SCNT/Pd-IPMC, especially the interface electrode, was greatly improved in comparison with the Pd-IPMC. The SCNT/Pd-IPMC included plentifully nanodispersed Pd particles inside the SCNT/Nafion hybrid layers, and the Pd nanoparticles combined with the SCNTs naturally. This resulted in thick composite interface electrodes, the thickness of which was identical to those of the SCNT/Nafion hybrid layers. The composite interface electrodes resulted from the deep penetration of [Pd(NH_3_)_4_]^2+^ and BH_4_^−^ into the SCNT/Nafion hybrid layers based on the high porosity and IEC. It can be deduced that this fabricating method can successfully create CNT/Pd composite interface electrodes with a strong synergistic effect by exploiting the SCNT/Nafion hybrid layer and nanodispersed Pd particles together. What is more, the widely penetrated Pd nanoparticles together with the SCNT contributed to the large surface areas, thereby accommodating more charges and increasing the capacitance of the IPMCs. This feature will facilitate the mass transport in IPMCs, which is a crucial factor in the fields of ionic EAPs (iEAPs), as well as sensors and fuel cells.

### 3.3. Electrochemical Properties of the IPMCs

**Figure 6:**
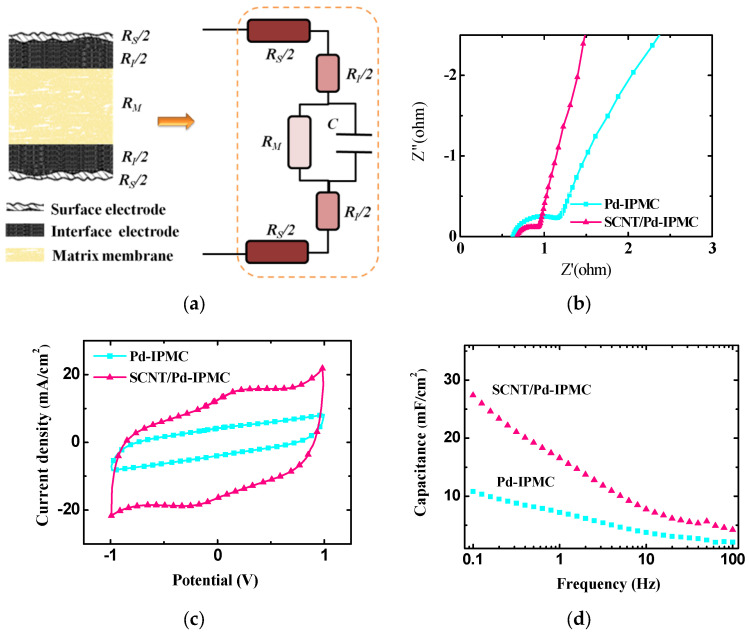
Electrochemical properties of the IPMCs. (**a**) Equivalent circuit of IPMC with SCNT/Pd electrode (*R_S_* is the surface electrode resistance, *R_I_* is the interface electrode resistance, *R_M_* is the membrane resistance, C is the capacitance); (**b**) Nyquist plot; (**c**) CV curves; (**d**) frequency dependence of the differential capacitance.

According to previous reports [14,16,17,38], the electrode and interlayer of IPMCs exhibit resistive-capacitive (R-C) properties under applied voltages. In the case of the interlayer, the IPMC system can be described by an RC-circuit, which corresponds to a coexistence of the internal ionic resistance and a formation of double layers, which determines the capacitive properties of the system (as shown in Figure 6a). The electrochemical properties of the IPMCs were studied using EIS and CV testing. The EIS test was carried out over a frequency range from 10^5^ to 0.1 Hz with an applied voltage of 50 mV, while the CV test was carried out over a potential range from −1.0 to 1.0 V at a scan rate of 50 mV/s.

The Nyquist plot in Figure 6b shows the real part of impedance (Z’ (f)) of the developed IPMCs. The impedance of the surface electrode, interface electrode, and inner Nafion layer is a critical factor determining the electrochemical behavior of IPMCs. The characteristic impedance at low frequencies is oriented by the ion diffusion process, while the characteristic impedance at high frequencies is oriented by the charge-transfer process [15,24,31]. Compared with the Pd-IPMC, the SCNT/Pd-IPMC showed much lower impedance. The decrease in impedance of the SCNT/Pd-IPMC was mainly attributed to the synergistic effect of the accelerated transport of hydrated cations and the larger interface electrode surface.

As the interface electrode between the surface electrode and inner Nafion layer can be viewed as an electrochemical capacitor, its capacitance reflects the ability to aggregate hydrated cations at the electrode–electrolyte interface, which strongly affects the electromechanical characteristics of IPMCs [51]. The CV curves of the Pd-IPMC and SCNT/Pd-IPMC are shown in Figure 6c. Obviously, the SCNT/Pd-IPMC preceded more superior capacitance and charge/discharge behaviors beyond the Pd-IPMC. The capacitance (C) of the IPMCs was calculated using the following equation:

(4) $$C=\frac{\left|I^{+}\right|+\left|I^{-}\right|}{2dV/dt}$$

where *I^+^* is the positive current density at 0 V, *I*^−^ is the negative current density at 0 V, and *dV/dt* is the scan rate. The capacitance value was 28.9 mF/cm^2^ for the SCNT/Pd-IPMC, which was over 3 times greater than that of the Pd-IPMC (8.05 mF/cm^2^). This indicates that a large number of charges can accumulate at the composite interface electrode surface to form a superior electrical double layer in the SCNT/Pd-IPMC. The differential capacitance of the IPMCs recorded by EIS testing is represented in Figure 6d, the trend of which was identical to that of the CV results. This implies a dramatically improved capacitance behavior of the SCNT/Pd-IPMC (27.4 mF/cm^2^ at 0.1 Hz) compared to that of the Pd-IPMC (10.8 mF/cm^2^ at 0.1 Hz).

To our knowledge, IPMC can be viewed as a parallel capacitor, the capacitance of which is in direct proportion to the permittivity and the contact area of the polymer/electrode interface, and in inverse proportion to the distance between the top and bottom electrodes [16,17,27]. Indeed, the enhanced capacitance demonstrates that the SCNT/Pd interface electrodes increased the contact area between the polymer and electrode, and also decreased the distance between the two electrodes. Moreover, the broadly penetrated Pd nanoparticles in the SCNT/Nafion layer improved the permittivity of the IPMC, which also contributed to increasing the capacitance.

These results indicate that the SCNT/Pd composite electrode with plentifully penetrated Pd nanoparticles and large interface areas provided the SCNT/Pd-IPMC with superior electrochemical behaviors, which are also expected to greatly improve the electromechanical behaviors.

### 3.4. Electromechanical Behaviors of the IPMCs

**Figure 7:**
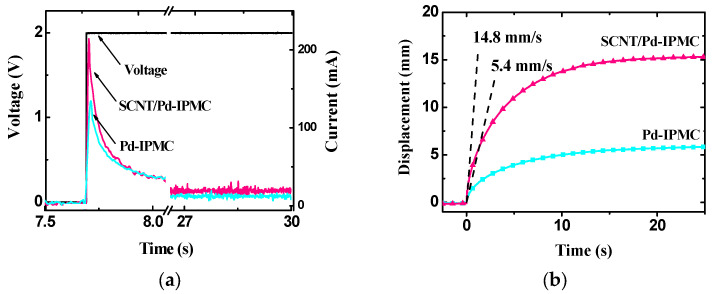
Electromechanical behaviors of the IPMCs. (**a**) Actuation current curves; (**b**) displacement curves under 2 V DC voltage in 25 s.

Figure 7 represents the electromechanical behaviors of the IPMCs. In the actuation current curves of both IPMCs under 2 V DC voltage (See Figure 7a), we can only observe the charging and discharging process. This indicates that the actuation mechanism of IPMCs is based on a double-layer electrostatic model [1,14,52]. The charging amount of hydrated cations at the interface electrode surfaces in the SCNT/Pd-IPMC (274 mC) was much larger than that of the Pd-IPMC (189 mC) by comparing via integrals and calculating the charging current curve for each IPMC. This is mainly considered to result from the broadly dispersed Pd nanoparticles, large surface area, and superior capacitance characteristics of the SCNT/Pd interface electrodes. We can imagine that the larger numbers of accumulated hydrated cations on the interface electrode surface will generate a larger driving force inside the SCNT/Pd-IPMC, which will result in superior actuation performances.

When submitting to applied voltages, the IPMCs deformed promptly. As expected, the SCNT/Pd-IPMC showed much greater actuation behaviors than those of the Pd-IPMC. The maximum displacement of the SCNT/Pd-IPMC under 2 V DC voltage in 25 s was 14.7 mm, which was about 2.5 times larger than that (5.92 mm) of the Pd-IPMC (as shown in Figure 7b). The deformation speed of the IPMCs was estimated from the initial slope of the displacement curves as the voltage was increased from 0 to 2 V. The maximum deformation speeds were 14.8 mm/s and 5.4 mm/s for the SCNT/Pd-IPMC and Pd-IPMC, respectively. Thus, the SCNT/Pd-IPMC responded much faster compared to the Pd-IPMC. To our knowledge, the displacement and deformation speed of the SCNT/Pd-IPMC are comparable with other solid-state iEAP actuators [25,38,45,52].

**Figure 8:**
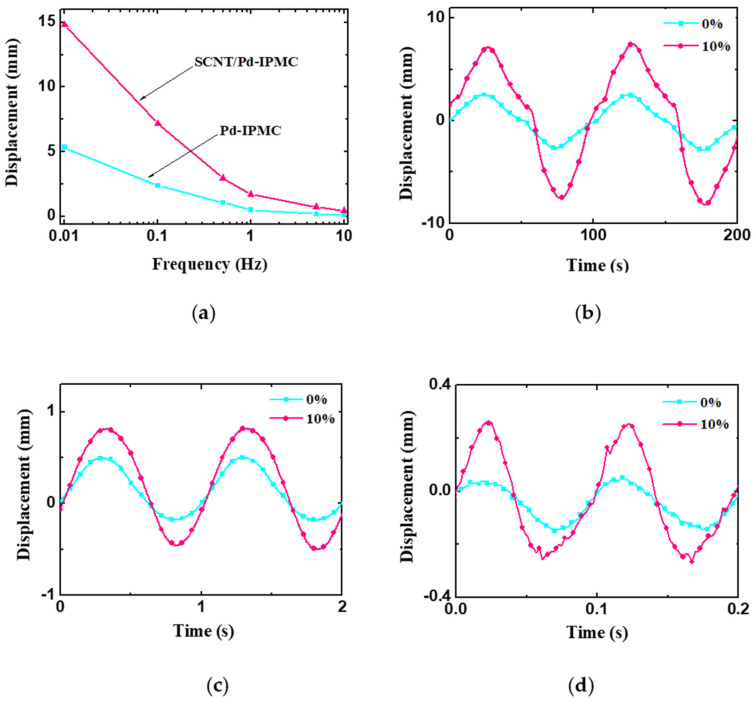
Actuation bending deformations of the IPMCs. (**a**) Harmonic response under ±2 V sinusoidal voltage; (**b**) displacement curves at 0.01 Hz; (**c**) displacement curves at 1 Hz; (**d**) displacement curves at 10 Hz.

The harmonic response of the IPMCs under ±2 V sinusoidal voltage is shown in Figure 8a, the trend of which is consistent with that of the deformation under DC voltages. Figure 8b–d represent the displacement curves at 0.01 Hz, 1 Hz, and 10 Hz, respectively. Notably, the peak-to-peak displacement decreased as the frequency increased, which implies an inverse correlation between the deformation and frequency. This is the same as the trend of the capacitance-frequency dependence of the IPMCs. The reduction in deformation as the frequency increases is considered to result from the decrease in the acting time of the applied voltage at a high frequency. Even so, the harmonic response of the SCNT/Pd-IPMC is much greater than that of the Pd-IPMC and that of SWCNT-Nafion IPMC [53]. This is mainly attributed to the formation of the SCNT/Pd interface electrodes, which effectively enlarged the available contact areas, and also shortened the hopping distance for the hydrated cations in the intermediate layers [54].

**Figure 9:**
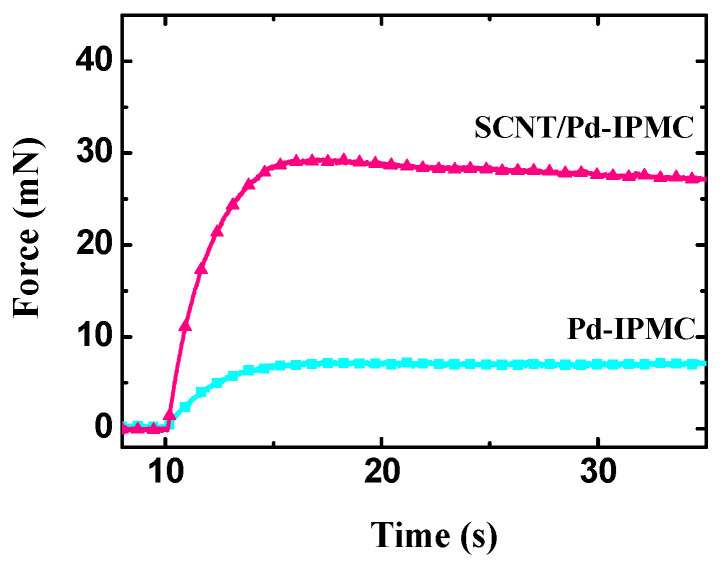
Blocking force curves under 2 V DC voltage in 25 s.

When a 2 V DC voltage was applied to the IPMCs for 25 s, the SCNT/Pd-IPMC output a blocking force up to 28.6 mN, which was almost 4 times as large as that of the Pd-IPMC (7.16 mN) (as shown in Figure 9). The blocking force of the SCNT/Pd-IPMC was much higher than those of most previous reported IPMCs [14,38,53,55]. In bending stiffness testing, the SCNT/Pd-IPMC showed a stiffness of 522.6 MPa, while the Pd-IPMC showed a stiffness of 187.9 MPa. The effective increase in stiffness of the SCNT/Pd-IPMC is attributed to the excellent mechanical strength and superior dispersity of the SCNTs and the plentiful penetration of the Pd nanoparticles in the Nafion matrix. Accordingly, the considerable increment in the mechanical stiffness of the SCNT/Pd-IPMC together with the superior electrochemical properties resulted in tremendous enhancement in its blocking force.

In order to comprehensively analyze the influence of the formation of SCNT/Pd interface electrodes on the actuation characteristics of the IPMCs, we calculated the volume work density (*W_D_*) from the displacement and blocking force using the following equation [56]:

(5) $$W_{D}=\frac{2d}{3ht}\frac{F\delta }{d^{2}+\delta^{2}}$$

where *d* is the measuring distance (m), *h* is the width (m), *t* is the thickness (m), *δ* is the maximum displacement (m), and *F* is the maximum blocking force (N) under 2 V DC voltage of the IPMCs. We also calculated the volume work density of IPMCs incorporated with SCNTs in electrolyte layers reported in our previous work [38] for comparison. The results are shown in Table 1. The SCNT/Pd-IPMC showed the highest volume work density of 5.83 KJ/m^3^, which was almost 10 times as large as that of the Pd-IPMC, and was also superior to those of the IPMCs incorporated with SCNTs in the electrolyte layers. This is because the high amount of SCNTs in the SCNT/Nafion hybrid layer greatly promoted the infiltration of nanodispersed Pd particles, and then resulted in composite interface electrodes with large surface areas and superior interfacial characterizations. In general, the actuation behaviors of the SCNT/Pd-IPMC have been significantly improved. This indicates that the formation of SCNT/Pd interface electrodes sufficiently enhanced the comprehensive actuation characteristics of the IPMC and simultaneously gave rise to a large bending deformation and output force as expected.

What is more, as the thickness of the SCNT/Pd interface electrode is identical to that of the SCNT/Nafion layer, and the electrochemical and electromechanical properties are mainly determined by the thickness of the interface electrode. It is expected that we can explore an actual requirement-oriented regulation method of the interface properties by adjusting the thickness of the SCNT/Nafion layer to improve the actuation behaviors (such as response speed, bending deformation, and blocking force) according to engineering application requirements, which work is currently being undertaken. The details will be presented and discussed in our forthcoming works.

## 4. Conclusions

In this contribution, we demonstrated a novel way to simultaneously enhance the deformation and output force of IPMCs by exploiting a synergistic effect of combining an SCNT/Nafion hybrid layer with nanodispersed Pd particles. The developed SCNT/Pd composite interfacial electrodes played a critical role in facilitating ion migration and charge injection in the IPMCs based on their relatively high IEC and large surface areas. The resulting IPMC exhibited significantly optimized electromechanical behaviors as expected, including about 2.5 times larger displacement, 3 times faster deformation speed, 4 times greater output force, and 10 times higher volume work density compared to those of the traditional Pd-IPMCs. The advantages of the SCNT/Pd-IPMCs will greatly facilitate their applicability as actuators, in particular for the development of artificial muscles.

The proposed method, which successfully developed the SCNT/Pd composite electrode in IPMCs, might present a new method of creative design for polymer/metal interfaces with excellent interface characteristics. In addition, the thickness of an SCNT/Pd composite electrode can be controlled by adjusting the thickness of the SCNT/Nafion layer. We expect that the specific preparation route of the composite electrode could build a new bridge between polymer and electrode design. We also expect to explore an actual requirement-oriented regulation method of the interface properties by adjusting the thickness of the SCNT/Nafion layer to improve the actuation behaviors according to engineering application requirements, which may have potential applications in supercapacitors, sensors, and fuel cells.

## Figures and Tables

**Table 1:** Volume work density values of IPMCs with SCNTs in SCNT/Pd composite electrode and SCNT/Nafion composite electrolyte.

IPMC	WD (KJ/m^3^)
Pure Nafion (0 wt% SCNT)	0.60
10 wt% SCNT in SCNT/Pd composite electrode	5.83
0.25 wt% SCNT in SCNT/Nafion composite electrolyte	0.79 ^a^
0.5 wt% SCNT in SCNT/Nafion composite electrolyte	1.67 ^b^

^a^ and ^b^ were calculated using the data reported in our previous work [38].

## Data Availability

Data are contained within the article.

## Supplementary Materials

The following are available online at https://www.mdpi.com/article/10.3390/polym14173494/s1, Figure S1: Schematic illustration for actuation mechanism of IPMCs, Figure S2: Schematic illustration for the chemical synthesis of SCNTs, Figure S3: The FT-IR spectrum of SCNTs and MWCNTs, and visual representations of the dispersity analysis of MWCNTs and SCNTs, Figure S4: TG curves of MWCNTs and SCNTs, and TG curves of the P-membrane, S2-membrane, and M-membrane, Table S1: Elemental analysis data of MWCNTs and SCNTs, Figure S5: Schematic illustration for the two-terminal connection in the electrochemical testing of IPMC samples.
