# Supplementary material for: Fabrication and Characterization of a Novel Smart-Polymer Actuator with Nanodispersed CNT/Pd Composite Interfacial Electrodes
Source: Polymers (Basel). 2022 Aug 26;14(17):3494. doi: 10.3390/polym14173494 (PMC9459883; doi:10.3390/polym14173494)
Supplement: Supplementary file 1 [file polymers-14-03494-s001.zip › polymers-1824014-supplementary.pdf]

polymers

MDPI

Supplementary Materials

Fabrication and Characterization of a Novel Smart-Polymer Actuator with Nanodispersed CNT/Pd Composite Interfacial Electrodes

Jie Ru [1,2], Dongxu Zhao [3,*], Zicai Zhu [4] and Yanjie Wang [2,*]

1 Key Laboratory of Green and Precise Synthetic Chemistry and Applications, Ministry of Education, School of Chemistry and Materials Science, Huaibei Normal University, Huaibei 235000, China
2 Jiangsu Key Laboratory of Special Robot Technology, Hohai University-Changzhou, Changzhou 213022, China
3 College of Mechanical and Electrical Engineering, Inner Mongolia Agricultural University, Hohhot 010018, China
4 School of Mechanical Engineering, Xi'an Jiaotong University, Xi'an 710049, China
* Correspondence: dxzhao611@imau.edu.cn (D.Z.); yjwang@hhu.edu.cn (Y.W.)

1. Schematic illustration for actuation mechanism of IPMCs

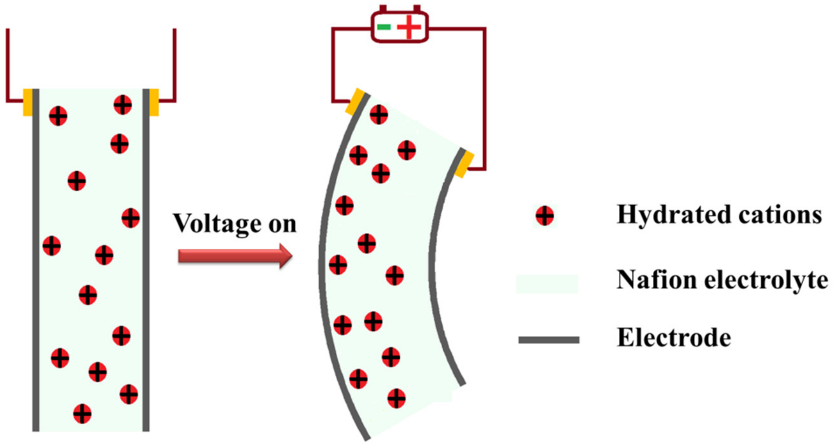

Figure S1. Schematic illustration for actuation mechanism of IPMCs.

2. Preparation of SCNT

SCNT was synthesized using the method reported by Hudson et al. [41]. The chemical reaction equation is presented in Figure S2.

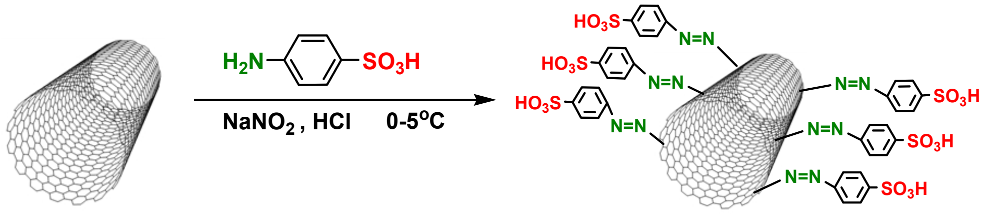

Figure S2. Schematic illustration for the chemical synthesis of SCNT.

The base process was as follows: 0.1g of MWCNT was added to 100 mL de-ionized water (is written water hereafter) and sonicated for 1 hr. Then 0.45 g of sodium p-aminobenzenesulfonate was put into the suspensions, and the pH value of the suspensions was adjusted to 1 using hydrochloric acid. After stirring for 10 min in an ice-water bath, 0.2 g of $NaNO_2$ in 2 mL water was added to the above mixture drop by drop. Hereafter, the reaction system was stirred for another 6 hrs with temperature at 0-5 °C and pH at 1. The product was filtrated out and washed with water and ethanol for several times and dried for 72 hrs in a vacuum-freeze dryer.

3. Characterizations of SCNT

3.1. Chemical composition and dispersity analysis of SCNT

SCNT was synthesized via diazotization coupling reaction of MWCNT and azo compounds in a strong acidic solution. The FT-IR spectrum of SCNT and MWCNT were represented in Figure S3a and the visual representations of the dispersity analysis were shown in Figure S3b.

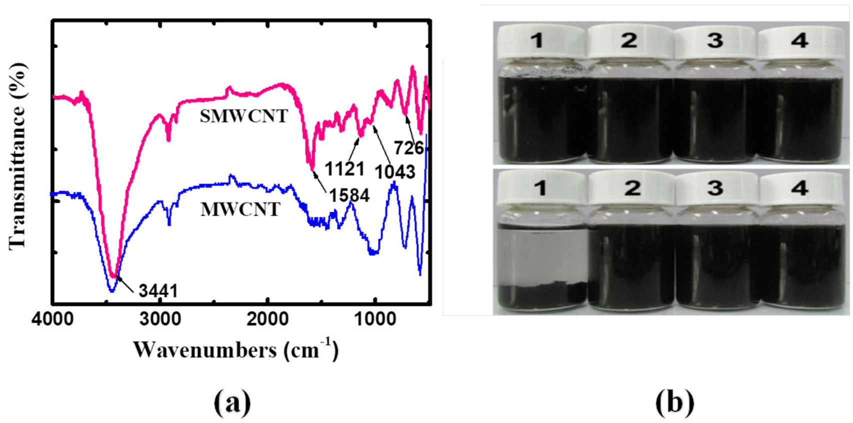

Figure S3. (a) The FT-IR spectrum of SCNT and MWCNT; (b) visual representations of the dispersity analysis (1 mg/mL) of MWCNT in water (1) and SCNT in water (2), EG (3) and Nafion solution (4) (the upper picture was taken just after sonication and the lower one was taken 24 hrs later).

The characteristic peaks of SCNT were confirmed by contrasting with those of MWCNT. The symmetric absorption peaks of the sulfonic acid groups ($-SO_3H$) were found at 1043 cm^{-1} and 1121 cm^{-1}, and the asymmetric absorption peak was found at 1584 cm^{-1}, which confirmed the existence of the $-SO_3H$ groups in SCNT after diazotization. The characteristic peaks of the benzene rings ($-C_6H_4-$) and the hydroxyl groups (-OH) were found at 726 cm^{-1} and 3441 cm^{-1}, respectively. It can be visually observed that the stretching vibrations at wavenumbers 3441 cm^{-1} appeared in both the spectras of MWCNT and SCNT, while the later was much stronger. In MWCNT spectra, the stretching vibrations were attributed to absorbed water which cannot be completely eliminated from MWCNT, while the stronger stretching vibrations of SCNT were caused by $-SO_3H$ group and absorbed water. In general, it can be confirmed that SCNT has been synthe-

sized successfully. What's more, it can also be supposed that the -SO_3H groups would provide the sites of hydrogen bonding between the SCNT and water. Therefore, the SCNT would have superior dispersity in water and Nafion matrix, which can be identified by the dispersity analysis of SCNT in Figure S3b.

The results of elemental analyses of both SCNT and MWCNT are summarized in Table S1. Notably, there were trace amount of nitrogen and sulfur in the pristine MWCNT. The emergence of nitrogen was attributed to the adsorption of nitrogen in MWCNT, while the emergence of sulfur was caused by the residual sulfur of the starting materials in preparing MWCNT. It can also be found that there were a considerable increase in nitrogen and sulfur content and a considerable decrease in C/N mass ratio and C/S mass ratio in SCNT compared to those in MWCNT, which were related to the incorporation of azobenzene-4-sulphonic acid into SCNT. The above results can powerfully confirm the successful synthesis of SCNT.

Table S1. Elemental analysis data of MWCNT and SCNT (C: carbon, H: hydrogen, N: nitrogen, S: sulfur).

	C (%)	H (%)	N (%)	S (%)	C/N ratio	C/S ratio
**MWCNT**	98.63	0.639	0.16	0.940	606.33	104.93
**SCNT**	92.24	0.888	0.36	1.614	258.53	57.15

3.2. TG of SCNT and Nafion membranes

Figure S4a represents the TG analysis of MWCNT and SCNT, and Figure S4b represents the TG analysis of the P-membrane, S2-membrane and M-membrame.

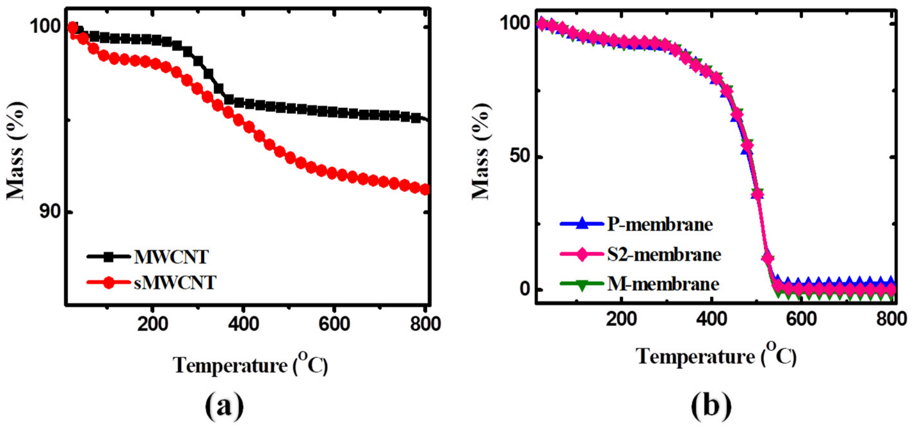

Figure S4. (a) TG curves of MWCNT and SCNT; (b) TG curves of the P-membrane, S2-membrane and M-membrane.

In Figure S4a, it can be found that the weight of SCNT nearly decreased by 10%, while the weight of MWCNT only decreased by 5%. In the curve of MWCNT, the small weight loss around 100 °C was caused by the evaporation of absorbed water molecules, while the weight loss between 220-400 °C was most likely

due to the elimination of amorphous carbon [40]. In comparison with MWCNT, the SCNT showed a slightly prominent weight loss, which was mainly caused by the decomposition of the grafted azobenzene-4-sulphonic acid and the elimination of amorphous carbon. The slow weight loss between 30-130 ^{o}C was also due to the evaporation of absorbed water molecules. The weight decreased dramatically in the range from 220 to 800 ^{o}C, which was caused by the decomposition of the side chains of SCNT and the elimination of amorphous carbon. The results can also indicate that the SCNT would be thermally stable in the membrane casting process.

The TG curves of the S2-membrane and M-membrane, with the same trend, indicate that the hybrid membranes are thermally stable upto ca. 300 ^{o}C, which is the same as that of the P-membrane. The gradual weight loss in the range between 30-300 ^{o}C was mainly due to the evaporation of water molecules. The thermal degradation of the membranes occurred in three stages: the initial stage between 300-400 ^{o}C was due to the desulfonation process of -SO_3H groups, while the second stage between 400-480 ^{o}C was caused by the decomposition of the branch chain from the polymer backbone, and the final stage between 480-550 ^{o}C was related to the decomposition of PTFE (Polytetrafluoroethylene) chain. It can be concluded that the hybrid membranes had the same thermal stability as that of the P-membrane.

4. Electrochemical characterizations

In the Cyclic voltammetry (CV) and electrochemical impedance spectroscopy (EIS) measurements for the IPMC specimens, the electrochemical VersaStudio (VersaSTAT 3, Princeton, USA) was used to obtain the CV curves and Nyquist plots. The CV measurements were carried out with potential range between -1.0 V and 1.0 V at a scan rate of 50 mVs^{-1}, and the EIS measurements were carried out over the frequency range from 100 kHz to 1 Hz with applied current of 100 mA. All the measurements were performed via the two-terminal connection with a specific area (illustrated in Figure S5) in DI water at room temperature. The size of IPMC strips for characterization was 35 mm × 5 mm × (360 ± 10) μm under hydrated conditions. In testing, IPMCs were connected from both side electrodes to the potentiostat terminals by pressing between two pieces of gold-plated copper plaque in order to eliminate the effect of electrode surface resistance.

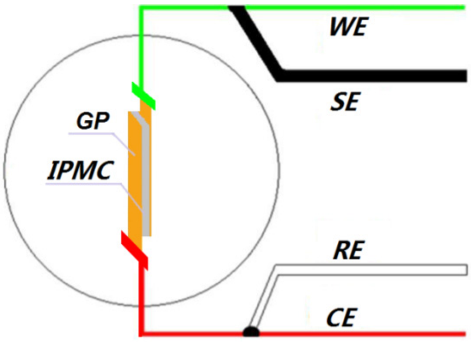

Figure S5. Schematic illustration for the two-terminal connection in the electrochemical test of IPMC samples, where WE is the working electrode of the VersaStudio, SE is the sensor electrode, RE is the reference electrode, CE is the counter electrode, and GP is gold-plated copper plaque.

Note: Reference number from article.
